# Supplementary material for: Low prevalence of current and past SARS-CoV-2 infections among visitors and staff members of homelessness services in Amsterdam at the end of the second wave of infections in the Netherlands
Source: PLoS One. 2023 Jul 25;18(7):e0288610. doi: 10.1371/journal.pone.0288610 (PMC10368265; doi:10.1371/journal.pone.0288610)
Supplement: S1 Table — (DOCX) [file pone.0288610.s001.docx]

**S.1. Table** Additional characteristics related to homelessness and lifestyle of
visitors of homeless services in Amsterdam, the Netherlands, May 2021.

|  | **Visitors (n=138) ^1^**  n(%) |
| --- | --- |
| **Duration of homelessness** (in years)  Median [IQR] | 3.0 [o.8-7.3] |
| **Current sleep location**  Independent living space  Permanently living with  acquaintances  Temporarily living with  acquaintances  Hotel  Squat, caravan, demolition site  Street  Emergency center, temporary  accommodation, night shelter  Housing facilities in homeless  shelters  Other (boat, tent) | 4 (3.0)  1 (0.7)  6 (4.4)  7 (5.2)  1 (0.7)  24 (17.8)  39 (28.9)  49 (36.3)  4 (3.0) |
| **Alcohol use**  No  Less than weekly  Weekly  Daily | 55 (41.0)  17 (12.7) 34 (25.4)  28 (20.9) |
| **Cannabis use**  No  Not regularly  Regularly | 51 (38.6)  27 (20.5)  54 (40.9) |
| **Hard drug use**  No  Not regularly  Regularly | 107 (81.1)  10 (7.6)  15 (11.4) |

Abbreviations: *IQR* interquartile range
^1^ Some variables report up to 6 missings.
