# Supplementary material for: Low prevalence of current and past SARS-CoV-2 infections among visitors and staff members of homelessness services in Amsterdam at the end of the second wave of infections in the Netherlands
Source: PLoS One. 2023 Jul 25;18(7):e0288610. doi: 10.1371/journal.pone.0288610 (PMC10368265; doi:10.1371/journal.pone.0288610)
Supplement: S2 Table — (DOCX) [file pone.0288610.s002.docx]

**S.2**. **Table** Daytime activities (potential COVID exposure) of visitors and staff members of homeless services in
Amsterdam, the Netherlands, May 2021.

| **Visitors (n=138)** | n(%) | **Staff members (n=53)** | n(%) |
| --- | --- | --- | --- |
| ***Activities in the past 7 days* ^*^** |  | ***Activities in the past 7 days*** |  |
| **Gone to work**  Never (0 times)  Sometimes (1-6 times)  At least daily (≥ 7 times) | 106 (78.5)  27 (20.0)  2 (1.5) | **Gone to work**  Never (0 times)  Sometimes (1-6 times)  At least daily (≥ 7 times) | 3 (5.7)  45 (84.9)  5 (9.4) |
| **Visited friends/family**  Never (0 times)  Sometimes (1-6 times)  At least daily (≥ 7 times) | 106 (79.1)  19 (14.2)  9 (6.7) | **Visited friends/ family**  Never (0 times)  Sometimes (1-6 times)  At least daily (≥ 7 times) | 19 (35.8)  34 (64.2)  0 |
| **Gone to a supermarket**  Never (0 times)  Sometimes (1-6 times)  At least daily (≥ 7 times) | 17 (12.7)  59 (44.0)  58 (43.3) | **Gone to a supermarket**  Never (0 times)  Sometimes (1-6 times)  At least daily (≥ 7 times) | 4 (7.5)  38 (71.7)  11 (20.8) |
| **Attended a religious service**  Never (0 times)  Sometimes (1-6 times)  At least daily (≥ 7 times) | 121 (90.3)  7 (5.2)  6 (4.5) | **Attended a religious service**  Never (0 times)  Sometimes (1-6 times)  At least daily (≥ 7 times) | 46 (86.8)  6 (11.3)  1 (1.9) |
| **Attended daytime activities at the shelter** ^a^  Never (0 times)  Sometimes (1-6 times)  At least daily (≥ 7 times) | 67 (50.0)  30 (22.4)  37 (27.6) | **Visited a restaurant/bar**  Never (0 times)  Sometimes (1-6 times)  At least daily (≥ 7 times) | 42 (79.2)  11 (20.8)  0 |
| **Gone to daytime community center (to**  **eat, shower, etc.)**  Never (0 times)  Sometimes (1-6 times)  At least daily (≥ 7 times) | 45 (33.6)  29 (21.6)  60 (44.8) | **Attended a party (e.g. birthday, wedding)**    Never (0 times)  Sometimes (1-6 times)  At least daily (≥ 7 times) | 47 (88.7)  6 (11.3)  0 |
| **Spent time on the street with others**  Never (0 times)  Sometimes (1-6 times)  At least daily (≥ 7 times) | 67 (50.0)  31 (23.1)  36 (26.9) | **Went outside** ^b^  Never (0 times)  Sometimes (1-6 times)  At least daily (≥ 7 times) | 12 (22.6)  15 (28.3) 26 (49.1) |
|  |  | **Care taking (e.g. informal care, providing groceries)**  Never (0 times)  Sometimes (1-6 times)  At least daily (≥ 7 times) | 43 (81.1)  10 (18.9)  0 |
|  |  | **Picked up medication or visited doctor**  Never (0 times)  Sometimes (1-6 times)  At least daily (≥ 7 times) | 46 (86.8)  7 (13.2)  0 |
|  |  | **Exercised in an organized context**  Never (0 times)  Sometimes (1-6 times)  At least daily (≥ 7 times) | 43 (81.1)  10 (18.9)  0 |
|  |  | **Exercised indoors (e.g. sports club, gym)**  Never (0 times)  Sometimes (1-6 times)  At least daily (≥ 7 times) | 51 (96.2)  2 (3.8)  0 |
|  |  | **Went to a recreational area (e.g. forest, beach, campsite)**  Never (0 times)  Sometimes (1-6 times)  At least daily (≥ 7 times) | 36 (67.9)  17 (32.1)  0 |
|  |  | **Went to the cinema, theater, concert, museum**  Never (0 times)  Sometimes (1-6 times)  At least daily (≥ 7 times) | 52 (98.1)  1 (1.9)  0 |

Abbreviations: *COVID-19* Coronavirus disease 2019. ^*^ Variables had up to 4 missing values. ^a^ Daytime activities at the shelter include
cleaning and cooking ^b^ Going outside includes going out for fresh air, to cycle, to run, to walk the dog, to play outside with their children.
