# Supplementary material for: Low prevalence of current and past SARS-CoV-2 infections among visitors and staff members of homelessness services in Amsterdam at the end of the second wave of infections in the Netherlands
Source: PLoS One. 2023 Jul 25;18(7):e0288610. doi: 10.1371/journal.pone.0288610 (PMC10368265; doi:10.1371/journal.pone.0288610)
Supplement: S3 Table — (DOCX) [file pone.0288610.s003.docx]

| **S.3**. **Table** SARS-CoV-2 previous testing, impact of the pandemic on daily activities for visitors and staff members of homeless services in Amsterdam, the Netherlands, May 2021. | | | |
| --- | --- | --- | --- |
|  | **Visitors  (n=138)**  n(%) | **Staff members (n=53)**  n(%) | **Total (n=191) ^1^**  n(%) |
| **Previously tested for SARS-CoV-2 infection**  Yes  No | 55 (41.0)  79 (59.0) | 37 (69.8)  16 (30.2) | 92 (49.2)  95 (50.8) |
| **Self-reported past infection**  No  Yes, and this has been confirmed  Yes, but this has not been confirmed  I do not know | 90 (66.7)  1 (0.7)  7 (5.2)  37 (27.4) | 24 (45.3)  8 (15.1)  13 (24.5)  8 (15.1) | 114 (60.6)  9 (4.8)  20 (10.6)  45 (23.9) |
| **Previously hospitalized with COVID-19  ^2^**  Yes  No | 0  8 (100) | 0  21 (100) | 0  29 (100) |
| **Impact of COVID-19 pandemic on daytime activities**  Daytime activities did not change  Daytime activities changed | 61 (45.5)  73 (54.5) | *NA* | *NA* |
| **Changes in daytime activities ^3^**  No more paid work  No or fewer daytime activities at shelter  Going outside less  More time at the shelter  Other  ^4^ | 24 (32.9)  11 (15.1)  22 (30.1)  27 (37.0)  24 (32.9) | *NA* | *NA* |

Abbreviations: *COVID-19* Coronavirus disease 2019; *SARS-CoV-2* Severe Acute Respiratory Syndrome
Coronavirus 2; *NA* not applicable.

^1^ Variables may have up to 4 missing values

^2^ among those with a previous positive SARS-CoV-2 test result

^3^ among those experiencing changes in their daytime activities since the COVID-19 pandemic

^4^ Other changes include being outside more, fewer leisure activities like going to the library, cinema, church, shopping, home earlier, fewer social contacts, less work.
