# Supplementary figures and images for: Low prevalence of current and past SARS-CoV-2 infections among visitors and staff members of homelessness services in Amsterdam at the end of the second wave of infections in the Netherlands
Source: PLoS One. 2023 Jul 25;18(7):e0288610. doi: 10.1371/journal.pone.0288610 (PMC10368265; doi:10.1371/journal.pone.0288610)

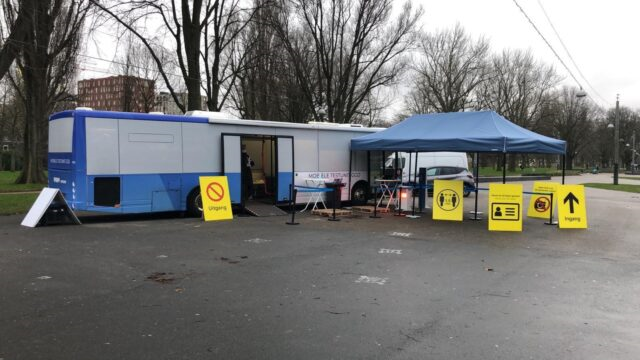


**S1 Figure**. The SARS-CoV-2 ‘testing bus’

Supplement: S1 Fig — (DOCX) [file pone.0288610.s004.docx]
