# Supplementary material for: Low prevalence of current and past SARS-CoV-2 infections among visitors and staff members of homelessness services in Amsterdam at the end of the second wave of infections in the Netherlands
Source: PLoS One. 2023 Jul 25;18(7):e0288610. doi: 10.1371/journal.pone.0288610 (PMC10368265; doi:10.1371/journal.pone.0288610)
Supplement: S2 Fig — (DOCX) [file pone.0288610.s005.docx]

*Low intention*

Combined vaccination intention

*Medium intention*

*High intention*

**S2 Figure.** Distribution of the combined score of two questions on intention to vaccinate
against SARS-CoV-2 among all study participants (n=168).
